# Increasing Family Planning Access in Kenya Through Engagement of Faith-Based Health Facilities, Religious Leaders, and Community Health Volunteers

**DOI:** 10.9745/GHSP-D-19-00107

**Published:** 2019-09-23

**Authors:** Allison Ruark, Jane Kishoyian, Mona Bormet, Douglas Huber

**Affiliations:** a Stellenbosch University Faculty of Medicine and Health Sciences, Stellenbosch, South Africa.; b Christian Health Association of Kenya, Nairobi, Kenya.; c Christian Connections for International Health, Alexandria, VA, USA.

## Abstract

Health facilities managed by faith-based organizations (FBOs) are important providers of health care in Kenya but provide only a small proportion of family planning services in the country. From 2013 to 2017, the Christian Health Association of Kenya (CHAK) implemented a project with 6 FBO-managed health facilities to increase voluntary family planning services in western Kenya, in partnership with religious leaders and community health volunteers (CHVs). The project aimed to build capacity of FBO-managed health facilities, increase religious leaders' knowledge of family planning, mobilize communities, improve family planning access and referrals for services, and advocate for improved family planning commodity security from the public sector. Project impact was evaluated using facility-level service statistics, project records and reports, and feedback from religious leaders and CHVs who implemented the project. Facility service statistics showed large increases in family planning visits. Phase 1 (2013–2014) was implemented at 2 health facilities, where client visits for family planning increased sixfold (from 705 to 4,286 visits) with tenfold increases seen in client visits for pills, intrauterine devices, and implants. In Phase 2 (2015–2017), the project was expanded to an additional 4 health facilities and total client visits for family planning nearly doubled (from 7,925 to 14,832 visits). During Phase 2, new client visits for implants increased threefold, making implants the most popular family planning method. Religious leaders who implemented the project reported reaching nearly 700,000 people with family planning messages and referring more than 87,000 clients to health facilities for family planning services. The religious leaders expressed confidence in the effectiveness of the project and in their role in enhancing access to voluntary family planning. Health facilities, religious leaders, and CHVs also reported multiple challenges to implementation, including inconsistent supply of family planning commodities from county health departments. This project demonstrates the potential of FBO-managed facilities and faith leaders to increase family planning demand and service provision, as well as the importance of coordination with the public sector to ensure supply of commodities and support for FBO-managed facilities.

## Abstract (precis)

The Christian Health Association of Kenya (CHAK) partnered with health facilities managed by faith-based organizations (FBOs), religious leaders, and community health volunteers to increase access to family planning in western Kenya. FBO-managed health facilities saw large increases in family planning uptake over the 5-year project, particularly for implants.

## BACKGROUND

Faith-based organizations (FBOs) are important providers of health care in many sub-Saharan African countries,^1^ yet available evidence suggests that FBO-managed facilities lag behind other health facilities in providing voluntary family planning services.^2^ In Kenya, FBOs own 16.5% of hospitals and 12.5% of all health facilities,^3^ but in 2014 only 2% of women reported obtaining contraception from FBO-managed facilities, and these facilities were less likely than public facilities and other privately managed facilities to offer family planning services.^4^ Catholic facilities offer only fertility awareness methods, and many Protestant facilities provide minimal family planning services.^2^ In 2010, only 69% of Kenyan FBO facilities offered family planning, compared with 97% of public facilities and 83% of private facilities, and only 29% of FBO facilities offered long-acting or permanent methods of family planning such as intrauterine devices (IUDs), implants, and male and female sterilization.^2^

FBOs in Africa are trusted by communities and thus well-placed to promote and provide family planning services,^5^ and religious leaders can similarly be influential advocates for family planning due to the respect and access they have within communities.^6^ Previous research has found that African religious leaders (both Christian and Muslim) understand the importance of family planning^7^^,^^8^ and are open to promoting family planning if provided with training and support.^9^ The power of religious leaders' pulpit can be seen in the fact that women who regularly attend religious services are more likely to use family planning,^7^^–^^10^ especially if their religious leaders have positive attitudes toward family planning and frequently speak about sexuality from the pulpit.^7^ Initiatives to expand family planning access through training staff of FBO-managed facilities and mobilizing religious leaders to raise awareness in their communities have succeeded in increasing family planning usage in Ghana,^11^ Ethiopia,^12^ Liberia,^5^ Rwanda,^13^ Uganda,^13^^,^^14^ and other African countries.^8^

This article presents an initiative of the Christian Health Association of Kenya (CHAK) to partner with FBO-managed health facilities and religious leaders to increase family planning access in the Nyanza and Western regions of Kenya. CHAK is a national FBO, founded in 1946, which comprises 24 hospitals, 57 health centers, 387 dispensaries, and 27 community-based health care programs owned by Protestant churches. These facilities and programs work in partnership with the Kenya Ministry of Health (MOH), county health departments, NGOs, development partners, the private health sector, and communities. CHAK has a central mandate of engaging religious leaders to improve health and a long-standing focus on family planning, including implementation of a World Bank-funded project in 2010 that trained pastors to educate parishioners about the value of family planning from the pulpit.^8^

## PROJECT CONTEXT

Nationally, Kenya's total fertility rate has declined from 4.9 births per woman in 2003 to 3.9 in 2014.^4^ Over the same period, contraceptive use has risen from 39% of married women in 2003 to 58% in 2014, with the largest gains seen in injectables and implants.^4^ Nationally, half of women of reproductive age do not want to have another child, and in Nyanza and Western regions this proportion is even higher (58% and 56%, respectively).^4^ Unmet need for family planning is also higher in these regions, at 23% in Nyanza and 21% in Western region, compared with 18% nationally (among married women of reproductive age).^4^

The project was implemented in Kakamega, Siaya, and Vihiga counties, which are contiguous counties in the Nyanza and Western regions of Kenya near Lake Victoria and the border with Uganda. These counties have a higher total fertility rate than the national average (Table 1). Four of 6 project sites were in Siaya County, which is notable for being among the 3 counties in Kenya with the lowest median age at first birth and for having one of the lowest levels of contraception use.^4^ CHAK chose to target these counties based on their high total fertility rate and unmet family planning need.

**TABLE 1.** Family Planning and Reproductive Health in Kakamega, Siaya, and Vihiga Counties, Kenya

	Kakamega County	Siaya County	Vihiga County	Kenya (national)
Estimated population (2009 census)	1,660,651	842,304	554,622	38,609,223
Total fertility rate	4.4	4.2	4.5	3.9
Age at first birth, years, median	19.8	18.7	20.3	20.3
Knowledge of contraceptive methods, %	100	100	100	99
Use of modern contraceptive methods, %	60	51	57	58
Use of any contraceptive method, %	62	55	60	63

Source: Kenya Demographic and Health Survey 2015,^4^ except for estimated population data taken from the 2009 Kenya Population and Housing Census.^15^

CHAK targeted 3 counties based on their high total fertility rate and unmet family planning need.

### Project Description

The ongoing project has a mandate to (1) build capacity of FBO-managed health facilities and community-based providers of family planning services, (2) sensitize Protestant, Catholic, and Muslim religious leaders, (3) mobilize communities, (4) improve family planning access and referrals from communities to health facilities, and (5) advocate for improved commodity security from MOH and county health departments. A particular goal of the project is to train and engage religious leaders to provide family planning information and referrals as part of their commitment to the health and well-being of families in their communities. Key project activities are highlighted in Table 2, in relationship to these project objectives.

**TABLE 2.** CHAK Family Planning Project Objectives and Activities in Kenya

Project Objective	Project Activities
1. Build capacity of FBO-managed health facilities and community-based providers of voluntary family planning services	CHAK built capacity through consultative meetings with participating health facilities and community stakeholder groups and developed capacity-building action plans. Health care workers and CHVs from each health facility were trained on provision of family planning services. CHAK provided technical assistance to family planning focal point persons through regular consultations and site visits, and CCIH provided long-distance technical assistance.
2. Sensitize religious leaders	Religious leaders were trained and given tools in support of referring clients and educating parishioners about family planning in churches, women's groups, and men's group, using materials developed by CHAK. Religious leaders participated in monthly meetings to support their family planning work.
3. Mobilize communities	Religious leaders and CHVs engaged in monthly dialogue days aimed at educating communities about family planning, dispelling myths, presenting family planning as consistent with Biblical principles, and explaining the benefits of family planning. CHVs distributed information, education, and communication materials to health facilities. CHVs and religious leaders engaged in ongoing community education and information sharing (often presenting together at the same event), including sensitization meetings in churches and communities.
4. Improve family planning access and referrals from communities to health facilities	Health facilities carried out quarterly outreach events to offer voluntary family planning services. CHVs carried out monthly community-based distribution of pills, condoms, and Cycle Beads. Religious leaders and CHVs referred clients to health facilities for family planning services.
5. Advocate for improved commodity security from MOH and county health departments	CHAK participated in family planning policy and planning meetings at county and national levels. CHAK supported health facility staff to attend quarterly county meetings to discuss family planning distribution to their facilities. Access to family planning commodities was ensured through strong collaboration with the county and national health management.

Abbreviations: CCIH, Christian Connections for International Health; CHAK, Christian Health Association of Kenya; CHV, community health volunteer; FBO, faith-based organization; MOH, Ministry of Health.

Phase 1 of the project was initiated in 2013 in 2 CHAK health facilities and their surrounding communities: Dophil Maternity and Nursing Home and Namasoli Health Center (see further details about facilities in Table 3). The objective was to enhance voluntary family planning performance at FBO-managed health facilities by mobilizing community health volunteers (CHVs) and religious leaders and ensuring adequate supplies of family planning commodities. In 2015, the project was expanded to 4 additional facilities and their surrounding communities (Phase 2): Kendu Adventist Mission Hospital, Kima Mission Hospital, Ng'iya Health Center, and Sagam Community Hospital (Table 3). All facilities equitably serve people of all faiths and religious traditions and offer on-site a range of modern family planning methods, including pills, injectables, IUDs, implants, male and female condoms, standard days method (using CycleBeads), Lactational Amenorrhea Method (LAM), and female sterilization. All facilities were chosen because they were located in areas with high need for family planning services and did not already have programs partnering with CHVs or religious leaders to increase family planning access.

**TABLE 3.** CHAK Project Health Facilities, Kenya

Name	Phases	Facility Level	County	Affiliation
Dophil Maternity and Nursing Home	1 and 2	3	Siaya	Nomiya Church
Kendu Adventist Mission Hospital	2	4	Siaya	Seventh Day Adventist
Kima Mission Hospital	2	2	Vihiga	Church of God
Namasoli Health Center	1 and 2	2	Kakamega	Anglican Church of Kenya
Ng'iya Health Center	2	3	Siaya	Anglican Church of Kenya
Sagam Community Hospital	2	4	Siaya	CHAK

Abbreviation: CHAK, Christian Health Association of Kenya.

The objective of the project was to enhance voluntary family planning performance at FBO-managed health facilities.

At the beginning of Phase 1, CHAK held meetings with the health facility management teams to share the project goals and objectives with them and ensure their engagement and support. Similar meetings were held with the management teams of the 4 facilities added during Phase 2. CHAK worked with health facility staff to carry out a situational analysis of past and current status of family planning services at the facilities and in the county. The situational analysis involved systematic review of data from the health facilities and from county Ministries of Health, and CHAK also interviewed hospital administrators and conducted focus groups with health care providers (nurses, clinical officers, and laboratory technicians), religious leaders, and CHVs. CHAK was able to identify challenges and bottlenecks to family planning access and develop a roadmap and implementation plan that defined desired outcomes and steps to achieving those outcomes.

Religious leaders and CHVs were tasked with implementing the project at the community level. They received project-branded hats and t-shirts to help identify them in their role as community educators, but they were unpaid. Religious leaders received a 3-day training focused on information about family planning, communication skills, and counseling skills. CHVs had already been trained by MOH but were not engaged in community-based distribution of family planning commodities prior to the project. They received a further 5-day training to equip them to counsel family planning clients and assist them in making informed family planning choices, distribute certain family planning commodities according to MOH guidelines (pills, condoms, and CycleBeads), and refer clients desiring other family planning methods to health facilities. Each CHV received a branded bag stocked with family planning commodities (pills, condoms, and CycleBeads) and a penis model to use in demonstrating male condom use. CHVs were also given funds for transport to allow them to fulfill their responsibilities.

Religious leaders and CHVs worked together to lead community dialogues about family planning. Before each dialogue, the leaders met to talk about family planning methods and address any questions or concerns about family planning (particularly among religious leaders). Religious leaders also invited CHVs to talk about family planning in forums such as church women's groups and youth groups. This approach has been so successful that CHAK has initiated additional projects to train religious leaders to educate their communities about hypertension and diabetes and to enlist their help in tuberculosis case finding.

Religious leaders and CHVs worked together to lead community dialogues about family planning.

Throughout Phases 1 and 2, CHAK held biannual stakeholder meetings with faith leaders, community gate keepers such as chiefs and community administrators, health workers, facility managers, CHAK managers, county officials, and county Ministry of Health staff. This engagement with stakeholders was crucial to garner support, avoid duplication of efforts, and ensure successful project outcomes. County health departments provided training materials, reporting tools and registers, and family planning commodities. Throughout project implementation, CHAK worked with county governments to ensure that the facilities received adequate supplies of family planning commodities. CHAK also supported facilities through offering on-the-job training for health workers (particularly training on long-acting family planning methods), supportive supervision, and mentoring.

CHAK received approval for the project from health facility administrators, health providers, community religious leaders, and religious leaders governing the health facilities. Health facilities approved use of facility-level data (which contained no personal identifiers) in evaluating the project. MOH and county health departments also approved and supported the project because it served to improve county family planning performance.

Funding for the project was provided by the David and Lucile Packard Foundation under the Africa Christian Health Associations Platform Family Planning Project in 2 separate grants, which accounts for the 2 phases of the project. Christian Connections for International Health (CCIH) collaborated closely with CHAK in the conceptualization, proposal development, and early implementation of the project, including baseline assessments at the health facilities. CCIH is an association of FBOs and individuals working in international health that has been a leader in advocating for family planning funding and promoting FBO involvement in family planning.^5^

## METHODS

In order to evaluate the impact of this project, we assessed multiple sources of data, including facility service statistics, project records and reports, and feedback from religious leaders and CHVs who implemented the project.

For Phase 1 (2013–2014), 2 of the authors (JK and DH) collected service statistics on new and return visits according to family planning method from the registers of Dophil Maternity and Nursing Home and Namasoli Health Center for 2012 (baseline prior to project implementation), 2013 (after project implementation), and 2014. All data collected from these health facilities were part of the standard MOH reporting system for counties, contained no identifying patient information, and provided a comparable and consistent data source before and during the project. In Phase 2 (2015–2017), the project added 4 additional health facilities (Table 2). Baseline data from the year prior to project implementation were not available for these 4 facilities. As for Phase 1, we collected data from facility registers on new and return visits according to family planning method, for all 6 facilities, for the period 2015–2017.

Throughout Phases 1 and 2, CHVs submitted monthly reports of how many clients they had provided with pills, condoms, and CycleBeads in households and communities. These clients were included in health facility data. In 2014, an unanticipated innovation led to another source of data with which to evaluate the pilot project. As well as providing family planning messages to their congregations and communities, religious leaders began making family planning referrals directly to CHVs and health facilities, an activity that had not been part of the original project design. The project thus asked religious leaders to begin reporting monthly, using a standardized form, the number of community members they had reached with family planning messages and the number of clients they had referred to health facilities and CHVs. Religious leaders and CHVs were provided with mobile phone-based reporting tools, but not all of them had phones or Internet access to support the technology; consequently, some chose to instead submit reports in hard copy. CHVs and religious leaders also submitted written feedback on the project as part of their monthly reports. The CHAK program coordinator (JK) made regular supervisory visits to the facilities to monitor the project and maintain an ongoing dialogue with religious leaders, CHVs, clinical staff, and the communities.

## FINDINGS

### Project Implementation and Perspectives From Religious Leaders and CHVs

In Phase 1 (2013–2014), 30 religious leaders and 60 CHVs were trained with updated family planning information, counseling, and referral skills. According to monthly reports submitted by religious leaders during Phase 1, they reached 5,154 clients in the Dophil catchment area and 7,198 clients in the Namasoli catchment area with family planning messages, and referred 450 and 988 clients to the 2 facilities, respectively. In Phase 2 (2015–2017), an additional 32 religious leaders and 52 CHVs were trained. By 2017, religious leaders had reached a total of 675,000 men and women with family planning information and had referred 85,810 clients to health facilities for family planning services.

By 2017, religious leaders had reached 675,000 people with family planning information and had referred 85,810 for family planning services.

Religious leaders and CHVs reported satisfaction with the project and confidence that it was benefiting the health and well-being of families in their communities. Religious leaders reported that they were comfortable in and enthusiastic about their role in providing education about family planning and referrals for family planning services. CHVs reported that community-based distribution reduced barriers to women accessing family planning services, and that working with religious leaders facilitated acceptance of their work by communities. Table 4 illustrates these perspectives with comments that religious leaders and CHVs submitted as part of their monthly reports.

**TABLE 4.** Perspectives From Religious Leaders and CHVs on the CHAK Family Planning Project, Kenya

	Quotes
Religious leaders	*The whole concept of family planning has always been viewed as a woman issue, that's the reason I decided to focus my messages towards men so that they can also get involved in family planning activities … [education] has changed the perception of many men about family planning.* —Female religious leader *I am happy that my community regards me as a family planning pastor. I am not ashamed to talk about family planning to the congregation wherever I get a chance. Many couples that I have encouraged to use family planning methods are now using the methods because as a teacher of the word of God they believe in my words. … I have been able to refer more than 100 clients to the health facilities for family planning methods … I have become a daktari meaning a doctor of family planning*. —Male religious leader *I referred one of my clients who had 7 children to the health center for an implant and her husband no longer quarrels her and insults her for “having many children like a rat.” I am happy when my clients are happy*. —Male religious leader
CHVs	*Before I started distributing pills to clients at my house, some of my clients used to have difficulty accessing the pills from the health facility since they had no approval from their husbands. Currently my clients can tell their husbands that they are coming to borrow something like salt or a pair of scissors from me and they get a chance to collect pills from me*. —Female CHV *I give my clients family planning pills during our monthly merry-go-round women's meeting [voluntary savings and loan association]. They don't have to travel to the health facility*. —Female CHV

Abbreviations: CHAK, Christian Health Association of Kenya; CHV, community health volunteer.

### Evidence of Project Impact From Health Facility Data

**FIGURE 1:**
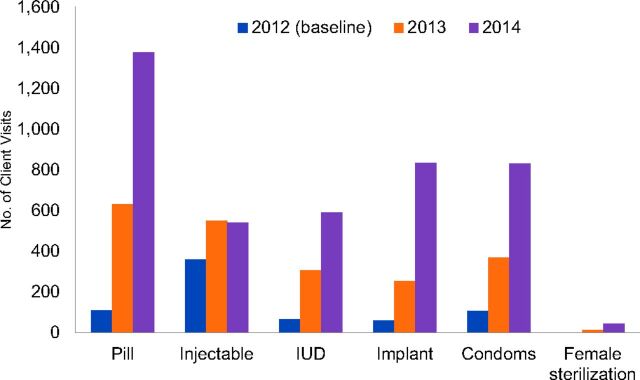
Total Client Visits^a^ by Family Planning Method at Dophil and Namasoli Health Facilities, Kenya, Phase 1 Abbreviation: IUD, intrauterine device. ^a^Includes new and returning client visits.

During Phase 1, Dophil and Namasoli saw large increases in family planning visits compared with the 2012 baseline (Figure 1). Total client visits for family planning increased threefold by 2013 (from 705 to 2,416) and sixfold by 2014 (to 4,286 visits). Rapid increases were seen for long-acting and reversible methods as well as user-dependent methods. Between 2012 and 2014, client visits increased approximately tenfold for pills (111 to 1,377 visits), IUDs (66 to 589 visits), and implants (61 to 835 visits). Dophil and Namasoli also saw large increases in visits for condoms (106 to 830 visits) and more modest increases for injectables (361 to 539 visits), while the number of sterilizations increased from 0 to 46. Voluntary sterilization for women generally required referral to a district hospital. Although male sterilization (vasectomy) was also available, it was not requested at either facility. In phase 2, these 2 facilities sustained high performance in project years 3–5, with Dophil seeing a continued increase in total family planning visits.

**FIGURE 2:**
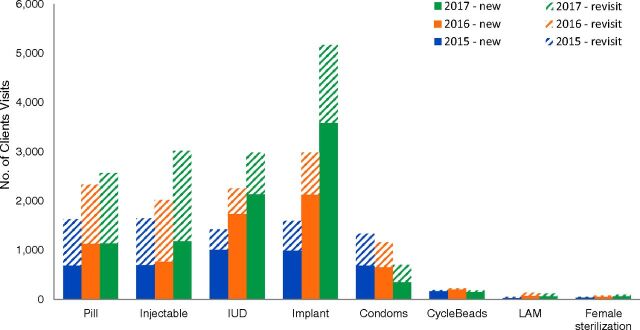
Number of Client Visits by New and Returning Visit and Family Planning Method for All 6 Health Facilities, Kenya, Phase 2 Abbreviations: IUD, intrauterine device; LAM, Lactational Amenorrhea Method.

In Phase 2, total client visits to the 6 health facilities for family planning nearly doubled, from 7,925 visits (2015) to 11,183 visits (2016) to 14,832 visits (2017). Data from Phase 2 also distinguished between new visits (which represent unique clients) and revisits (Figure 2). New client visits for implants more than tripled (997 to 3,588 visits), and implants were the most popular method in absolute terms (29% of all client visits). New client visits for IUDs more than doubled (1,018 to 2,142), new client visits for injectables nearly doubled (699 to 1,191), and new visits for pills increased by 66% (691 to 1,146 visits). Revisits for commodities such as pills and injectables stayed steady or increased over the period 2015 to 2017, suggesting that many women were continuing with the same family planning method. In contrast, the number of clients seeking condoms decreased by nearly half, which may indicate that these women were shifting to other family planning methods. The remaining methods were sought by only a small number of women (fewer than 200 per year for CycleBeads and LAM in 2017 and fewer than 100 per year for female sterilization); therefore, changes in demand for these methods should be interpreted with caution. According to feedback from health facilities, low uptake of fertility awareness methods (LAM and Standard Days Method using CycleBeads) may be attributable in part to health care workers placing greater emphasis on other methods.

In Phase 2, total client visits to the 6 health facilities for family planning nearly doubled.

According to health center staff, some returning visits were scheduled follow-up visits and others were initiated by clients who had experienced side effects, had questions, or had other needs for ongoing care. Injectables are effective for 3 months, meaning women using injectables should have returned 4 times per year. However, the number of revisits for injectables in 2016 and 2017 (the second and third years of Phase 2) suggests that not all new users of injectables were returning to the same facilities 4 times the following year. Revisits for injectables in 2016 and 2017 were only 2 to 3 times the number of new visits for injectables the previous year, consistent with typical discontinuation rates for injectables. This finding suggests that some women discontinued injectable contraceptives, while others may have obtained injections at facilities not included in this study. Similarly, data for pill revisits suggest that some women discontinued pill usage. Clients were typically given a 3-cycle supply of pills at each visit, but sometimes only 1 or 2 cycles of pills were provided if supplies were short, which may have contributed to some women discontinuing usage.

The number of revisits for IUDs was smaller than for pills and injectables, as expected. Women receiving IUDs were scheduled for 1-month and 3-month follow-up visits. The copper-bearing CuT 380A, which is effective for up to 12 years, was the most common IUD provided. Implants were primarily 3-year devices (Implanon or Nexplanon) and less commonly 5-year devices (Jadelle), and women receiving implants were scheduled for a 1-month follow-up visit after insertion. The number of revisits among women using implants was relatively high, compared with revisits among women using IUDs. Condoms were distributed in variable numbers, depending on available stock and client request, but the fact that a majority of visits for condoms were new visits (rather than revisits) suggests that women were not regularly obtaining condoms from these facilities as a primary means of contraception.

**FIGURE 3:**
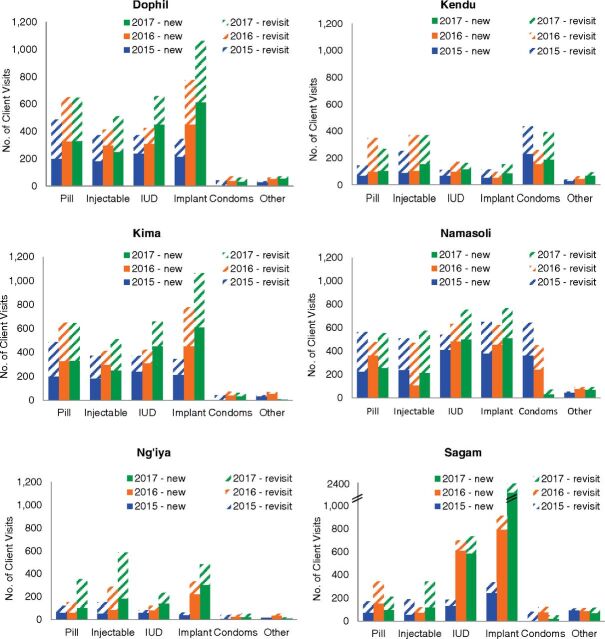
Number of Client Visits by New and Returning Visit and, Family Planning Method, and Facility, Kenya, Phase 2 Abbreviation: IUD, intrauterine device. Note: “Other” includes CycleBeads, Lactational Amenorrhea Method (LAM), and female sterilization.

**FIGURE 4:**
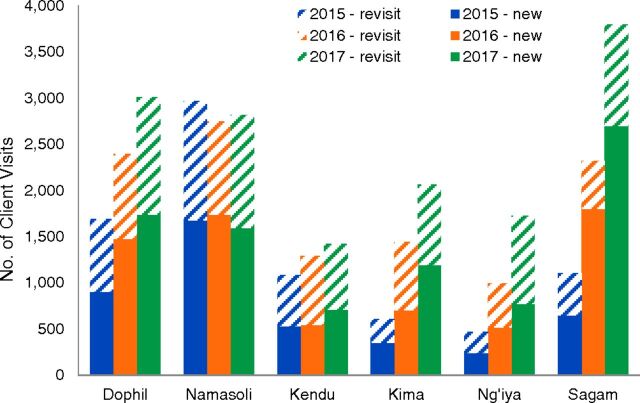
Number of Client Visits by New and Returning Visit and Facility, Kenya, Phase 2

As shown in Figure 3 and Figure 4, considerable variation existed between facilities in total client visits for family planning and in the popularity of various family planning methods. For example, more than half of new client visits for implants in 2017 were at Sagam Community Hospital, which was likely attributable to this facility conducting community outreach events twice per month, which was more than other facilities. In addition, the high numbers of implants distributed at Sagam may have been due to the high quality of counseling available at this facility (which is a teaching hospital) because good counseling can be a key factor in women deciding to use long-acting reversible methods such as implants. Clear increases in family planning visits were seen at all facilities between 2015 and 2017, with two exceptions. While Namasoli Health Center did not see a large increase in client visits for family planning during Phase 2, this facility had already seen visits increase substantially during Phase 1, from 384 in 2012 to 2641 in 2014. Kendu Adventist Mission Hospital saw little or no increases for most family planning methods over Phase 2, which may be due to several unique features of this facility. Kendu is a level-4 hospital located near other lower-level health facilities, and members of the community may have chosen to obtain family planning at those lower-level facilities rather than at a hospital. In addition, Kendu also restricted the family planning commodities it gave to CHVs with the justification that these commodities were in short supply at the facility. Other facilities saw new client visits approximately double (Dophil Maternity and Nursing Home), triple (Kima Mission Hospital and Ng'iya Health Center), or quadruple (Sagam Community Hospital) between 2015 and 2017.

Considerable variation existed between facilities in total client visits for family planning and in the popularity of various methods.

### Challenges Faced in Project Implementation

Whereas health facility data indicate that the project was highly successful in increasing family planning access, CHAK also noted a number of challenges to implementation faced by religious leaders, CHVs, and health facilities. These challenges are described in Table 5, along with solutions that were identified and/or implemented. The various challenges faced at multiple levels of the project illustrate the complexities of implementing such a project, even one that was highly successful according to multiple sources of data.

**TABLE 5.** Challenges Faced and Solutions Implemented by the CHAK Family Planning Project, Kenya

Challenges Faced	Solutions
**By religious leaders**	
Criticism from community members that it was inappropriate for religious leaders to talk about family planning and that they were straying from their mission to preach the word of God	Religious leaders received training to equip them as family planning educators and used relevant verses from the Bible and Quran to support their calling to educate people on health issues and promote family health through family planning.
Contraceptive myths and misconceptions, opposition to family planning based on religious beliefs	Religious leaders discussed facts of family planning in order to dispel myths and misconceptions, and used WHO materials as well as verses from the Bible and Quran to address opposition to family planning.
Technical questions from family planning clients or potential clients that religious leaders could not answer	Religious leaders referred such clients or potential clients to CHVs or health facilities, invited health facility staff or CHVs to speak about family planning during religious services and other community events.
Male Muslim leaders not able to reach women with family planning messages	Female Muslim religious leaders were recruited to conduct outreach to women.
**By CHVs**	
Stock-outs of family planning commodities (pills, condoms, and CycleBeads)	CHVs referred clients to health facilities for these commodities.
Hostility towards family planning, particularly the idea of not having more children (sometimes reinforced by religious leaders)	CHVs emphasized that the goal for family planning is to space births and limit family size to what the family wants and can care for, but not necessarily to stop having children.
Skepticism towards particular family planning methods, including beliefs that they are ineffective, harmful, or cause negative side effects	CHVs tried to combat myths and misconceptions, such as sterilization can cause cancer, family planning leads to weakness that makes women unable to work, and various methods are ineffective.
Referred clients not visiting the health facility due to lack of time and money or lack of support from husbands	CHVs conducted community outreach, including counseling couples about the benefits of family planning.
**By health facilities**	
Staff turnover, particularly of nurses trained on LARCs, such as implants and IUDs	Actions included on-the-job training and mentorship of new staff, improving work environment (such as through ensuring that commodities and supplies are available), and recognition of staff who perform well.
Commodity shortages and stock-outs, particularly during strikes at government facilities (3-month doctors' strike in 2016–2017 and 5-month nurses' strike in 2017), which led to increased demand for family planning services at FBO-managed facilities	Grant funds were used to purchase “buffer stock” (200 IUDs and 300 implants were purchased during Phase 2), and coordination with county health departments was undertaken to maintain adequate stock without need for project-purchased commodities (as achieved by Dophil and Namasoli).

Abbreviations: FBO, faith-based organization; CHAK, Christian Health Association of Kenya; CHV, community health volunteer; IUD, intrauterine device; LARC, long-acting reversible contraceptive; WHO, World Health Organization.

## DISCUSSION

The rapid and sustained increase in family planning uptake by the 6 participating health facilities over the course of project implementation indicates that FBOs can substantially expand services to help address unmet need for family planning in Kenya. Data on facility visits, as assessed in this study, have been found to be a fairly accurate proxy for trends in contraceptive prevalence.^16^ Thus, we see strong evidence for concluding that contraceptive prevalence rose in populations served by this program. We also note that religious leaders reported referring many more clients for family planning services than visited the project facilities for family planning services. Religious leaders referred 85,810 clients for family planning services in Phase 2, but the 6 facilities reported only 33,940 client visits for family planning over the same period. While some clients may not have followed through with the referral, others may have sought family planning at non-project facilities.

Implants were the most frequently requested family planning method and had the largest increase in demand. This high demand for implants is consistent with analysis showing that large increases in implant use across sub-Saharan Africa since the early 2000s are driving increased modern contraceptive use in the region.^17^ By the mid-2010s, Kenya had the highest prevalence of implant use among 17 sub-Saharan African countries for which such data were available, with the percentage of married women using implants increasing from 2% to 18% between 2008 and 2016.^18^

This project demonstrates that religious leaders can effectively reach large numbers of people in their communities and congregations with family planning messages, reinforcing previous findings that people have a high degree of trust in FBOs and religious leaders and that religious leaders are thus well placed to address the unmet need for family planning.^6^ Religious leaders also became active in referring family planning clients to CHVs and health facilities, a response that was initiated by the religious leaders themselves and not anticipated in the project design. CHAK's collaboration with religious leaders began by identifying common ground and building trust through continuous dialogue and sensitivity to faith leaders' perspectives and needs. Also crucial was empowering religious leaders through capacity building, training, family planning updates, and information, education, and communication materials to support their communication. Finally, continued engagement with religious leaders required continual interaction and support, as well as appropriate recognition and expressions of appreciation for their effort.

This project demonstrates that religious leaders can effectively reach large numbers of people with family planning messages.

CHVs were instrumental in bridging the gap between communities and health facilities. Their effectiveness depended on appropriation training, provision of family planning commodities, and motivation from health facility staff and their religious leaders. Monthly meetings between CHVs and health facility staff provided an opportunity for updates, problem solving, and commodity supply. At times CHVs also participated in church services to provide health and family planning messages.

County officials were also an integral part of the project, participating in launch and training workshops as well as providing commodities. In some cases, county officials also allowed staff who were paid by the MOH to be seconded to FBO facilities. Project results were included in county reporting, and county officials thus viewed the performance of FBO projects and facilities as part of county efforts to strengthen services. CHAK is now expanding this model of collaboration between FBO-managed health facilities and counties to other regions of the country. The challenges faced by health facilities in procuring family planning commodities from county health departments demonstrate the need for continuing coordination with the public sector, especially given the reality of constrained government resources for family planning and inconsistent supply of commodities. Continued advocacy, engagement, and good relationships with county officials are necessary to support staffing of health facilities and training of staff, as well as procurement of materials and commodities.

Procuring family planning commodities was a recurring challenge for this project and is a common problem across Africa, especially for FBO-managed facilities.^18^ A study of FBO-managed facilities in 13 African countries found that more than half had faced stock-outs of one or more reproductive health products (including contraceptives) in the past 3 months, requiring staff to implement creative solutions such as finding other sources of commodities and purchasing and storing buffer stock.^19^ The CHAK project adopted such strategies, procuring buffer stocks of contraceptives (particularly IUDs and implants) in both Phase 1 and Phase 2 of the project to ensure that lack of commodities would not limit performance.

Procuring family planning commodities was a recurring challenge and is a common problem across Africa, especially for FBO-managed facilities.

Besides the challenges described, we also note several limitations in the available data and interpretation of those data. Because the project had no control group, we cannot know to what degree factors external to the project and project facilities affected family planning uptake at those facilities. Stock-outs of family planning commodities at other facilities may have increased demand for family planning at the project facilities, and the health worker strikes during Phase 2 of the project may have had the same effect. Furthermore, no baseline data are available for the 4 facilities added in Phase 2, meaning that while data from 2015 to 2017 show an increase in family planning visits beginning in the first year of Phase 2, they cannot answer how family planning uptake changed from pre-implementation to the first year of Phase 2 implementation. The increase in family planning uptake for 2015–2017 likely underestimates the impact of the project in Phase 2.

### Recommendations

We offer the following recommendations for similar programs attempting to engage religious leaders and FBO-managed facilities for rapid and sustainable increases in voluntary family planning. In addition, CHAK is willing to share expertise and any tools developed for the project, including training materials and reporting forms, and would welcome visits to the health facilities that implemented the project.

#### Sensitively Engage Religious Leaders

The project should ensure that religious leaders are appropriately invited and adequately trained to participate in the project, and it should include religious leaders of all faith traditions if possible. Adequate training includes up-to-date information on methods of family planning and addressing common misconceptions held by the community and religious leaders themselves. In this project, health facility staff issued invitations to Christian and Muslim leaders, which were well received. Also critical was giving religious leaders time to understand the training they received on family planning, ask questions, and discuss their new knowledge as well as their role in the project with their congregations. This collaborative process of “internal advocacy” was crucial to religious leaders (who are mostly men) having the support of their faith communities in taking on the role of family planning advocate.

#### Ensure Ownership of the Project by Various Stakeholders

As noted, stakeholder engagement meetings were critical to ensure that government officials, faith leaders, health facilities, and other key stakeholders concurred with the project's goals and put their support behind the project in practical ways. The project also found other ways to increase stakeholder investment in the project. Providing CHVs and religious leaders with branded bags, t-shirts, and hats built morale and gave them a sense of ownership and commitment to the project. Communicating with CHVs, religious leaders, and health facilities about the project's progress at regular meetings, and sharing data from all health facilities with them, helped them to see the “big picture” of project impact and increased their investment in the project's success. These meetings also gave stakeholders an opportunity to discuss how the project might improve.

#### Design a Realistic Monitoring and Evaluation Strategy and Data Management Plan

A carefully designed monitoring and evaluation strategy is crucial for documenting progress toward project goals and should be implemented from the beginning of the project. Effective data management can be challenging in an environment in which electricity and telecommunications can be erratic. This project found that CHVs and religious leaders were often unable to use electronic reporting tools designed for mobile phones (via Internet or SMS) because they lacked funds to purchase data for their phones and also required training in use of these electronic tools. The project did not have funds for such training or to purchase mobile phone data for religious leaders and CHVs, so religious leaders and CHVs submitted paper reports to health facilities. Projects should be careful to design a monitoring and evaluation system that is realistic in the project environment.

## CONCLUSION

We believe this project provides an effective, scalable, and sustainable model for engaging with FBO-managed facilities, CHVs, and religious leaders to increase family planning demand and services in sub-Saharan Africa. These 3 partners formed a vital triangle of effective service provision, and the linkages between them enhanced performance and results. This study makes a particular contribution in identifying lessons learned for mobilizing religious leaders as effective promoters of voluntary family planning and facilitating access to services through referrals. Even given recent increases in family planning usage within sub-Saharan Africa, considerable unmet need remains. Greater support for the extensive service delivery infrastructure provided by FBOs, and effective engagement of religious leaders, can make a significant impact on addressing the unmet need for family planning in Africa.

## Notes

**Peer Reviewed**

**Funding:** David and Lucile Packard Foundation.

## References

[1] Kagawa RC Anglemyer A Montagu D. The scale of faith-based organization participation in health service delivery in developing countries: systemic review and meta-analysis. PLOS One. 2012;7(11):e48457. 10.1371/journal.pone.0048457 23152775 PMC3495941

[2] Barden-O'Fallon J. Availability of family planning services and quality of counseling by faith-based organizations: a three country comparative analysis. Reprod Health. 2017;14(1):57. 10.1186/s12978-017-0317-2. 28482905 PMC5423000

[3] Luoma M Doherty J Muchiri S. Kenya Health System Assessment 2010. Bethesda, MD: Abt Associates Inc.; 2010. https://www.hfgproject.org/wp-content/uploads/2015/02/Kenya-Health-Systems-Assessment-2010.pdf. Accessed July 29, 2019.

[4] Kenya National Bureau of Statistics. Kenya Demographic and Health Survey 2014. Nairobi, Kenya: National Bureau of Statistics; 2015. https://dhsprogram.com/pubs/pdf/fr308/fr308.pdf. Accessed August 21, 2019.

[5] Barot S. A common cause: faith-based organizations and promoting access to family planning in the developing world. Guttmacher Policy Rev. 2013;16(4):18–23. https://www.guttmacher.org/gpr/2013/12/common-cause-faith-based-organizations-and-promoting-access-family-planning-developing. Accessed August 21, 2019.

[6] Allison A Foulkes E. Engaging Faith Leaders in Family Planning. Washington, DC: World Vision US; 2014. https://www.worldvision.org/wp-content/uploads/2017/03/Engaging-Faith-Leaders-in-Family-Planning.pdf. Accessed July 29, 2019.

[7] Yeatman S Trinitapoli J. Beyond denomination: the relationship between religion and family planning in rural Malawi. Demogr Res. 2008;19(55):1851–1882. 10.4054/DemRes.2008.19.55. 20463916 PMC2867343

[8] Aylward L Friedman N. Faith and International Family Planning. Washington, DC: World Faiths Development Dialogue; 2014. https://universalaccessproject.org/wp/wp-content/uploads/2015/06/UNF-WFDD-Family-Planning-Full-Report-FINAL.pdf. Accessed July 29, 2019.

[9] Alikali M. The attitudes and activities of pastors and faith leaders in Zimbabwe on the use of family planning methods among their members. Christian J Glob Health. 2017;4(2):66–74. 10.15566/cjgh.v4i2.188

[10] Agadjanian V. Religious denomination, religious involvement, and modern contraceptive use in southern Mozambique. Stud Fam Plann. 2013;44(3):259–274. 10.1111/j.1728-4465.2013.00357.x. 24006073 PMC4604208

[11] Duah J Yeboah P. Family planning practice among Christian health service providers in Ghana: a case study. Christian J Glob Health. 2017;4(2):80–86. 10.15566/cjgh.v4i2.175

[12] Olson DJ Piller A. Ethiopia: an emerging family planning success story. Stud Fam Plann. 2013;44(4):445–459. 10.1111/j.1728-4465.2013.00369.x. 24323662

[13] VanEnk L Kasyaba R Kanani PB Tumwesigye T Cachan J. Closing the gap: the potential of Christian Health Associations in expanding access to family planning. Christian J Glob Health. 2017;4(2):53–65. 10.15566/cjgh.v4i2.164

[14] Muhirwe LB. Approaches for integrating primary health care in reproductive health programs in Uganda. In: Olivier J Wodon Q, eds. The Role of Faith-Inspired Health Care Providers in Sub-Saharan Africa and Public-Private Partnerships. Vol 1. Washington, DC: The World Bank; 2012:134–142. http://documents.worldbank.org/curated/en/851911468203673017/pdf/762230v10WP0Fa0Box374365B000PUBLIC0.pdf. Accessed July 29, 2019.

[15] Kenya National Bureau of Statistics. The 2009 Kenya Population and Housing Census. Nairobi, Kenya: National Bureau of Statistics; 2010. https://s3-eu-west-1.amazonaws.com/s3.sourceafrica.net/documents/21195/Census-2009.pdf. Accessed August 21, 2019.

[16] Magnani RJ Ross J Williamson J Weinberger M. Can family planning service statistics be used to track population-level outcomes? Glob Health Sci Pract. 2018;6(1):93–102. 10.9745/GHSP-D-17-00341. 29467167 PMC5878083

[17] Jacobstein R. Liftoff: the blossoming of contraceptive implant use in Africa. Glob Health Sci Pract. 2018;6(1):17–39. 10.9745/GHSP-D-17-00396. 29559495 PMC5878070

[18] Ali M Farron M Ramachandran Dilip T Folz R. Assessment of family planning service availability and readiness in 10 African countries. Glob Health Sci Pract. 2018;6(3):473–483. 10.9745/GHSP-D-18-00041. 30213877 PMC6172130

[19] Metzger AM Bormet M. Pharmaceutical stockouts: problems and remedies for faith-based health facilities in Africa. Christian J Glob Health. 4(2):19–29. 10.15566/cjgh.v4i2.130

